# Effect of Heartfulness Meditation on Oxidative Stress and Mindfulness in Healthy Participants

**DOI:** 10.7759/cureus.62943

**Published:** 2024-06-23

**Authors:** Yogesh Patil, Kishore Sabbu, Ranjani B Iyer, Sanjana T Philip, Alphonso Armila Nadhar, Kapil S Thakur, Poonam Kadu, Mansee Thakur

**Affiliations:** 1 Medical Biotechnology, Mahatma Gandhi Mission (MGM) School of Biomedical Sciences, MGM Institute of Health Sciences, Navi Mumbai, IND; 2 Medical and Research Centre, Kanha Medical Services, Kanha Shanti Vanam, Hyderabad, IND; 3 Curriculum Development for Heartfulness, Heartfulness Institute, San Ramon, USA; 4 Heartfulness Meditation Centre, Heartfulness Institute, Panvel, IND

**Keywords:** mental health, oxidative stress, nitrate, malondialdehyde (mda), psychometric evaluations, heartfulness meditation

## Abstract

Background: Mental health issues are a major cause of poor life outcomes. Heartfulness (HFN) meditation is recommended for stress management and daily awareness. Although studies have shown that HFN can improve burnout and well-being, the biological mechanism underlying oxidative stress markers in a healthy human is unclear.

Objective: The purpose of this study was to determine whether HFN meditation benefits mindfulness responses and also to examine the impact of HFN meditation on oxidative stress in healthy individuals.

Methods: This prospective study involved 60 healthy individuals aged 18-24, divided into experimental and control groups, and implemented an HFN meditation intervention over 12 weeks. Both groups' serum malondialdehyde and serum nitrate levels were examined before and after the intervention. Additionally, psychometric evaluations concerning mindfulness and experiential avoidance were conducted utilizing scales such as the Mindful Attention Awareness Scale (MAAS), Five-Facet Mindfulness Questionnaire (FFMQ), Self-Compassion Scale (SCS), and Experiential Avoidance (EA).

Results: Following a three-month intervention period, serum malondialdehyde (MDA) levels in the experimental group did not show a significant increase, whereas in the control group, there was a significant increase (p < 0.000). Conversely, serum nitrate levels in the experimental group exhibited a significant increase (p < 0.05). Additionally, psychological stress decreased, as indicated by various questionnaire tools such as MAAS, FFMQ, SCS, and EA, with mindfulness showing an increase. However, a decrease in EA was seen.

Conclusion: Heartfulness meditation has a positive impact on both mindfulness and oxidative stress. This suggests that consistent, long-term participation in HFN meditation could enhance mental health and foster overall well-being.

## Introduction

Nowadays, a lot of people suffer from anxiety and stress as a result of their fast-paced, complicated lifestyles that include a lot of expectations in their personal and professional lives. Persistent, long-term stress can exacerbate or create physical and mental health issues, including early mortality [1]. Untreated mental health issues among children and teenagers are associated with negative impacts on their physical health, academic performance, and social interactions, leading to increased tendencies towards anxiety, depression, self-harm, and suicidal behaviors [2]. Depression is a common mental disorder seen across all age groups, including children and adolescents. While biomedical research has advanced in line with technology, it does not appear to have undergone a comprehensive transformation. Additionally, several illnesses have emerged and are currently incurable. Consequently, efforts have been made to improve therapy and quality of life using complementary and alternative medicines.

Many psychological therapies have been developed to address these problems. In recent years, there has been a remarkable emphasis on interventions based on meditation, a rigorous spiritual and psychophysical practice. Meditation helps to attain self-awareness, relaxation, calmness, and a clear, quiet, and steady mind by establishing a link between the mind and body [3]. Several techniques of meditation, such as loving-kindness, body scanning, Zen, mindfulness, heartfulness (HFN) meditation, breathing, and concentration meditation, have been used since ancient times and are rooted in several religions and traditions [4]. In line with earlier research on the beneficial effects of psychological well-being on the human lifespan, meditation has progressively drawn attention from scientists as a means of encouraging aging healthily [5].

These mental health issues can also cause oxidative stress and psychological disorders. A psychological trait can be identified with the term “mindfulness.”. One of the most often used instruments for evaluating mental health is mindfulness, which can be measured using the Mindful Attention Awareness Scale (MAAS), the Five Facet Mindfulness Questionnaire (FFMQ), and the Self-Compassion Scale (SCS) [6, 7]. Oxidative stress is considered to be one of the main causes of many diseases. It is also associated with a range of mental ailments, such as depression, anxiety disorders, and bipolar disorder [8]. Malondialdehyde (MDA) is considered by many experts to be a crucial component of oxidative biomarkers. Nitric oxide (NO) is another oxidative stress biomarker that undergoes various chemical processes in the body, and its ultimate products are nitrate and nitrite [9, 10]. Better lifestyle choices and reducing oxidative stress can influence telomerase activity, prevent telomere shortening, delay the onset of age-associated diseases, and increase lifespan. This can be done through engaging in meditation, breathing exercises, yoga, or any other spiritual activity [4, 11]. Although some well-conducted studies have shown a connection between oxidative stress and mental health problems, it is unclear how these conditions are causally related to one another. Understanding the precise nature of the connection between cellular oxidative stress and emotional stress will require a great deal of effort and study.

Heartfulness meditation is a kind of meditation that employs three primary methods: 1) meditation, 2) rejuvenation, and 3) prayer. According to Sylapan et al., these methods are meant to enlarge and purify consciousness and awareness of oneself [11, 12]. The benefits of HFN meditation on both physical and mental health outcomes have been demonstrated; however, the mechanisms behind its effects on oxidative stress biomarkers have not been thoroughly studied. Therefore, in our study, we are focusing on HFN meditation to explore the influence of experience avoidance and compassion, as well as the impact of HFN meditation on oxidative damage and psychological stress.

## Materials and methods

Aim

This study aimed to determine whether HFN meditation benefits mindfulness responses and examine the impact of HFN meditation on oxidative stress in healthy individuals.

Study design

The research was a 12-week prospective cohort investigation that took place in April 2023 and ended in December 2023. It was decided to use a convenience random sample technique to find 60 healthy, matched volunteers with one active control condition (sham meditation) and one intervention condition (guided HFN meditation). This is a double-arm, randomized, controlled study. Volunteers were contacted via email by the principal investigator (PI) to find out their interest in being a part of this research. Enrolled individuals shared a similar lifestyle and were matched according to age (±2 years) and gender. The study was initiated after ethical clearance on February 15, 2023 (MGM/DCH/IEC/059/23) was obtained from the Institutional Ethics Committee of Mahatma Gandhi Mission (MGM) Dental College & Hospital (IEC-MGMDCH). All of the participants provided their informed consent before participating in the study.

Participants

Eligibility Criteria for the Participants

**Figure 1:**
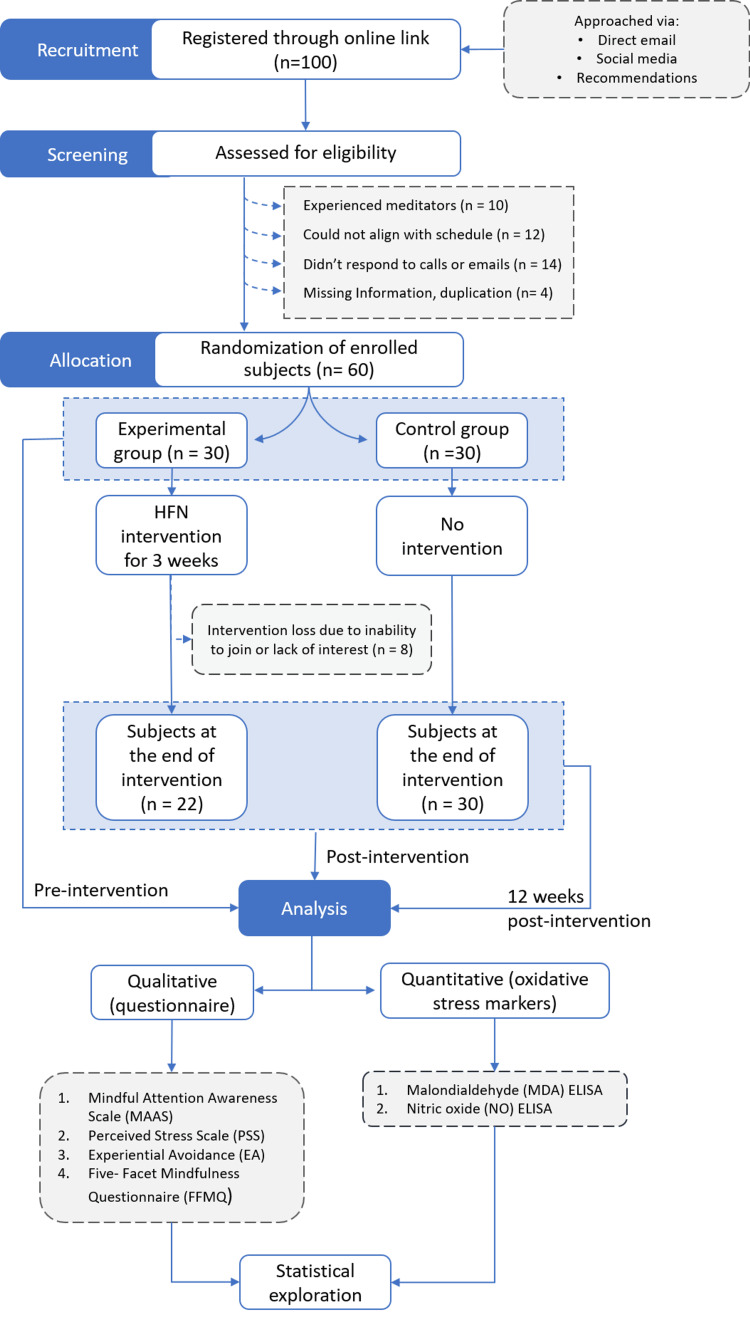
A flow diagram outlining the study process HFN: heartfulness; ELISA: enzyme-linked immunosorbent assay

The key inclusion criteria were male or female aged 18-24 years without a history of bacterial, viral, or fungal infection or any infections in the last two months. None of the participants in this study had any prior experience practicing any form of meditation techniques. Participants with a history of psychosis, bipolar disorder, major depressive disorder, seizure disorder, mental retardation, autoimmune disorders, immune deficiency disorders, neurological diseases, and pregnant women were excluded from the study. The study flowchart is showcased in Figure 1.

Sample Size Calculation

According to G*Power Software (version 3.1, Heinrich-Heine-Universität, Düsseldorf, Germany), a sample size of 60 participants (30 meditators and 30 non-meditators) was determined for the effect size of 0.8, and 80% power for Type I error; α = 0.05, by applying the t-test family to compare the difference between two independent means (two groups: study and comparative) [13]. 

Randomization and Blinding

Computer-generated randomization was used for randomization and the participants were blinded to the study.

Intervention

A certified trainer in this field taught the members of the experimental group how to lead HFN meditation for 30 minutes on each of the three consecutive days [12, 14]. This meditation routine was conducted once a day in the morning. These participants practiced the meditation technique once daily on all working days (online through the HeartsApp application version 4.1.5 (Heartfulness Institute, Hyderabad, India) and offline) for 12 weeks, and on holidays, they used HeartsApp software. Every week, they would come together for meditation at the Heartfulness Meditation Centre in New Panvel, Navi Mumbai, India. The participants were also advised to attend a 15-minute rejuvenation session, during which they were instructed to envision their challenges, tension, and mass leaving their bodies as smoke or vapor. Instead of worrying about the items they were getting rid of, the participants were instructed to disregard them. The participants were also given guided audio clips to follow HFN core practices (meditation, rejuvenation, and bedtime prayer) every day for 12 weeks.

Outcome measures

Qualitative Assessment Using Questionnaire Tools

Survey instruments like the SCS, EA, FFMQ, PSS, and MAAS were utilized to show how meditation affected several psychological dimensions. The participants of both control and experimental groups were examined for these variables (questionnaires) for pre-intervention as well as post-intervention as follows:

Perceived Stress Scale: The PSS was used to measure stress. This 10-item questionnaire is designed to measure subjective stress. The scale calculates how stressful a certain circumstance is rated. A five-point Likert scale, with 0 representing never and four representing very often, is used to rate the response items. Higher scores indicate higher levels of perceived stress. The scores range from 0 to 40. The PSS has proven to be reliable in undergraduate college samples [15, 16].

Mindful Attention Awareness Scale: The MAAS, established by Brown and Ryan in 2003, is a 15-item assessment tool developed to gauge the extent of open or receptive awareness and attention to present experiences, a fundamental aspect of dispositional mindfulness. The MAAS questionnaire includes questions such as I find it difficult to stay focused on what’s happening in the present; I find myself doing things without paying attention; and I snack without being aware that I’m eating. This scale has undergone validation using samples from community members, college students, and cancer patients, demonstrating strong psychometric properties. Response choices span from one (almost always) to six (almost never) [6, 7].

Five Facet Mindfulness Questionnaire: This questionnaire comprises 39 items assessing the five facets of mindfulness, which include aspects such as observing (e.g., when I’m walking, I deliberately notice the sensations of my body moving), describing (e.g., I’m good at finding words to describe my feelings), action and awareness (e.g., when I do things, my mind wanders off and I’m easily distracted), non-judging of inner experience (e.g., I criticize myself for having irrational or inappropriate emotions), and non-reactivity of inner experience (e.g., I perceive my feelings and emotions without having to react to them). Higher scores on the scale reflect greater self-reported mindfulness skills. The items are evaluated using a Likert-type scale ranging from one (never or very rarely true) to five (very often or always true) [17].

Self-Compassion Scale: Self-compassion and well-being are related to each other. Self-compassion refers to a kind-hearted way of relating to one’s failures, weaknesses, and disappointments. To evaluate self-compassion, the following aspects are evaluated: self-kindness (e.g., I try to be understanding and patient towards those aspects of my personality I don’t like), self-judgment (e.g., I’m disapproving and judgmental about my own flaws and inadequacies), common humanity (e.g., I’m disapproving and judgmental about my own flaws and inadequacies), isolation (e.g., when I’m feeling down, I tend to feel like most other people are probably happier than I am), mindfulness (e.g., when something painful happens, I try to take a balanced view of the situation), and over-identified (e.g., when I fail at something important to me, I become consumed by feelings of inadequacy). For example, higher SCS scores have been associated with higher levels of goal success, pleasure, and life satisfaction; they have also been associated with healthier physiological responses to lower levels of depression, anxiety, and stress [18].

Experiential avoidance: Experiential avoidance is measured using a questionnaire called- The Acceptance and Action Questionnaire-II (AAQ-II). This measures several psychological flexibilities of an individual fused with thoughts and avoiding feelings. The AAQ-II is a questionnaire tool that includes seven items that assess a person’s experiential avoidance. The AAQ-II items are assessed using a seven-point Likert-type scale, ranging from one (never true) to seven (always true) [19].

Quantitative assessment

Measurement of Blood Biomarkers Related to Oxidative Stress

Blood samples were drawn by venipuncture from the median cubital vein. A total of 3 ml of blood was collected in plain vacutainer tubes from all the participants. The serum sample was immediately separated after coagulation, and the samples were stored at -80⁰C until assay. The samples were taken from each of the groups, viz., the control and intervention groups. The oxidant-antioxidant defense mechanism was evaluated by using MDA and NO as oxidative stress biomarkers. Serum nitrate was measured using Cayman's nitrate/nitrite colorimetric assay (Cayman Chemical, Ann Arbor, MI) and serum MDA using the Elabscience kit (Elabscience, Houston, TX) [8, 10].

Statistical Analysis

We used the Mann-Whitney test to analyze differences in outcomes between the experimental and control groups. Spearman's rank correlation was also used to test the correlation between various variables. IBM SPSS Statistics software for Windows, version 24 (IBM Corp., Armonk, NY) was utilized to calculate these statistics. All the data were presented as the mean value along with the standard deviation (SD).

## Results

Demographic profile

The sociodemographic characteristics, comprising age, gender, and sleeping hours, of the total 60 participants who participated in the study are presented in Table 1. The age group was between 18 and 24 years old. There were 42 (70%) participants in the age group 18 to 20 (70%); 12 (20%) in the age group 20 to 22 (20%); and six (10%) in the 22-years and above age group. The total average age of participants was 19.20 years, with an SD of 1.39. Among these, the majority of the participants were female (N = 45, 75%), and male participants were 15 (25%). Fifty-six participants had study hours of one to <5 hours (93.33%), four participants had study hours of five to <10 hours (6.66%), and no participant had study hours of less than one hour or more than 10 hours. The PSS served as a precise tool for assessing personal stress levels and was widely used to understand how various circumstances affect emotions and stress levels. It was observed that the participants had a moderate level of stress (18.79 ± 1.21). According to the PSS assessment, the results of this present study show an average value of 18.79 before the three-month intervention. After the intervention, the average value decreased to 17.18 (showing a mean difference of 1.6), indicating a statistically significant reduction in perceived stress (P-value = 0.006).

**Table 1:** Sociodemographic characteristics of the participants

Characteristics	
Age (years)	
Mean (SD)	19.20 (1.39)
Range	
Age groups (years), n (%)	18-24
18 to <20	42 (70)
20 to < 22	12 (20)
>= 22	6 (10)
Gender: n (%)	
Male	15 (25)
Female	45 (75)
Study hours: n (%)	
<1	0
1 to <5	56 (93.33)
5 to <10	04 (6.66)
>10	0

Qualitative assessment

Mindful Attention Awareness Scale

Mindfulness was evaluated among participants from both groups by using the MAAS questionnaire. There was no significant difference observed between the experimental group and control group participants pre- and post-intervention, as shown in Table 2.

**Table 2:** Mindful Attention Awareness Scale (MAAS) results of the participants

Variable	Experimental group (mean, SD)			Control group (mean, SD)		
MAAS	Pre-intervention	Post-intervention	P-value	Pre-intervention	Post-intervention	P-value
	49.148(16.9)	54.346(13.94)	0.242	51.407(13.08)	46.28(10.65)	0.183

Five-Facet Mindfulness Questionnaire

The FFMQ includes aspects such as observing, describing, action, and awareness; non-judging of inner experience; and non-reactivity of inner experience. Higher scores on the scale reflect greater self-reported mindfulness skills. The FFMQ total score increased in the experimental group (122.77±16.54) post-intervention as compared to the control group (113.96±24.57). However, this increase was significant only in the cases of observation and non-reactivity (Table 3).

**Table 3:** Five Facet Mindfulness Questionnaire (FFMQ) results of the participants

Variable	Experimental group (mean, SD)			Control group (mean, SD)		
	Pre-intervention	Post-intervention	P-value	Pre-intervention	Post-intervention	P value
Observing	22.814(6.079)	29.652(4.43)	0.000	24.6(5.49)	24.045(6.03)	0.736
Describing	22.841(5.44)	22.521(5.033)	0.819	23.6(4.68)	19.454(4.69)	0.22
Acting with awareness	24.629(8.55)	26.260(5.47)	0.319	24.68(7.30)	22.181(7.23)	0.247
Non-judging of inner experience	23.333(8.14)	23.043(5.43)	0.770	23.12(6.75)	20.00(6.25)	0.057
Non-reactivity of inner experience	18.111(5.13)	22.00(3.44)	0.001	21.44(4.28)	20.272(3.82)	0.324
FFMQ	111.222(23.176)	123.478(14.27)	0.058	117.44(16.46)	105.95(16.66)	0.014

Self-Compassion Scale

The SCS is an indicator of HFN meditation towards compassion for oneself and gratitude. Higher scores reflect a greater potential for self-compassion. This increase in score was significantly observed in the experimental group between pre-and post-intervention, except for the parameter of mindfulness (Table 4).

**Table 4:** Self-Compassion Scale (SCS) results of the participants

Variable	Experimental group (mean, SD)			Control group (mean, SD)		
	Pre-intervention	Post-intervention	P-value	Pre-intervention	Post-intervention	P-value
Self-kindness	4.962(2.21)	8.57(1.21)	0.000	6.370(1.46)	3.740(1.32)	0.000
Self-judgment	6.481(1.77)	8.346(1.17)	0.002	6.259(2.08)	6.48(1.17)	0.689
Common humanity	6.888(1.68)	8.346(1.29)	0.002	6.444(1.74)	6.2(1.16)	0.804
Isolation	6.888(1.68)	8.576(0.96)	0.000	5.703(1.90)	5.72(1.77)	0.930
Mindfulness	8.259(2.08)	8.692(1.26)	0.390	6.444(1.81)	7(1.44)	0.271
Over-identified	6.962(1.773)	8.538(1.39)	0.003	5.888(1.68)	5.8(1.72)	0.742
SCS	40.44(7.11)	51.076(2.71)	0.000	34.481(5.88)	37.56(5.01)	0.061

Experiential Avoidance

The Acceptance and Action Questionnaire-II (AAQ-II) tool is a seven-item self-report to evaluate experiential avoidance. Elevated scores on the AAQ-II indicate increased EA and immobility; conversely, lower scores suggest greater degrees of acceptance and action. Therefore, after 12 weeks of intervention, in the experimental group, the EA scores decreased significantly, as shown in Table 5.

**Table 5:** Experiential Avoidance results of the participants AAQ-II: Acceptance and Action Questionnaire-II

Variable	Experimental group (mean, SD)			Control group (mean, SD)		
AAQ-II	Pre-intervention	Post-intervention	P-value	Pre-intervention	Post-intervention	P-value
	25.370(11.08)	19.692(7.44)	0.047	24.962(8.95)	29.32(9.97)	0.066

Qualitative and biochemical assessments

Serum Nitrate

**Figure 2:**
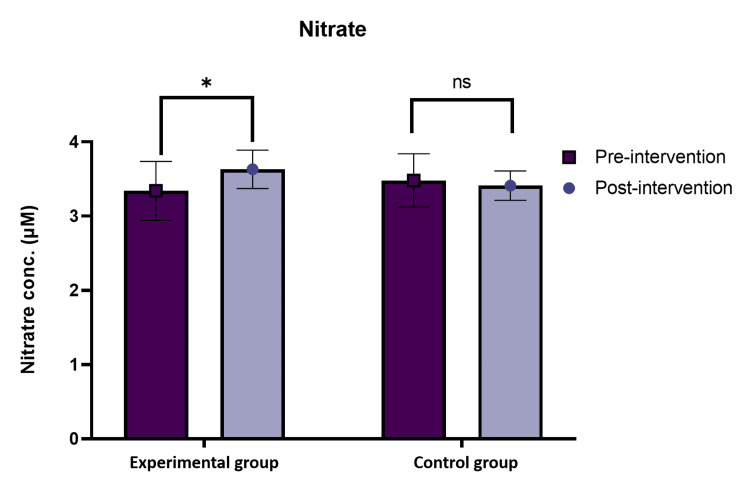
Oxidative biomarkers of serum nitrate levels (µM) were significantly increased in the experimental group between pre- and post-test. conc.: concentration

The serum nitrate was checked in both the control and experimental groups. It was observed that there was a significant increase in nitrate in the experimental group as compared to the control group (Figure 2).

Serum MDA

**Figure 3:**
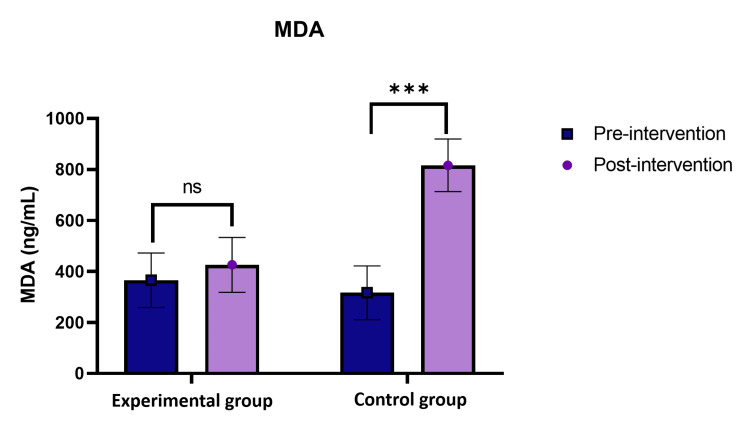
Oxidative biomarkers of serum MDA levels (ng/mL) in participants were significantly increased in the post-control group. MDA: malondialdehyde

The serum MDA was checked in both the control and experimental groups (Figure 3). It was observed that there was a significant increase in MDA in the control group as compared to the experimental group.

Correlation of Mindfulness (FFMQ) With Other Questionnaires

**Figure 4:**
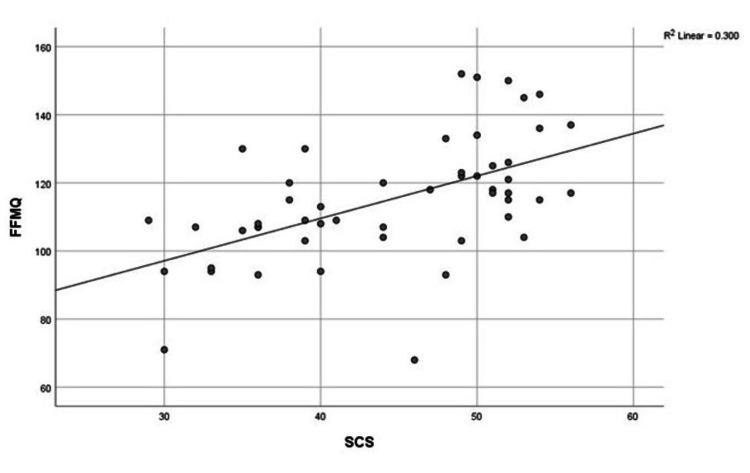
Correlation between FFMQ and SCS FFMQ: Five Facet Mindfulness Questionnaire; SCS: Self-Compassion Scale

**Figure 5:**
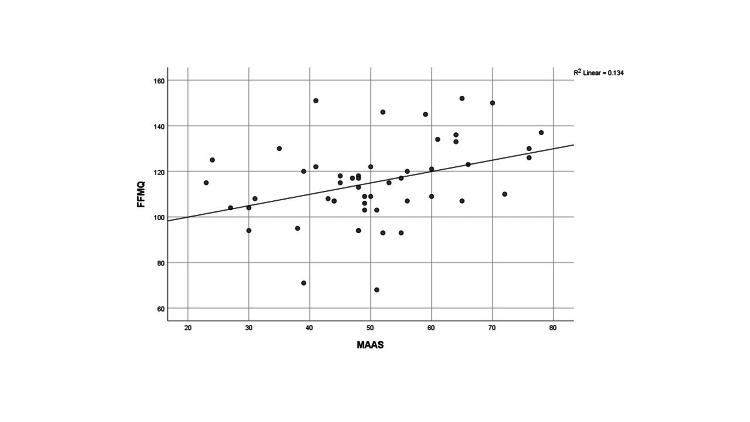
Correlation between FFMQ and MAAS FFMQ: Five Facet Mindfulness Questionnaire; MAAS: Mindful Attention Awareness Scale

**Figure 6:**
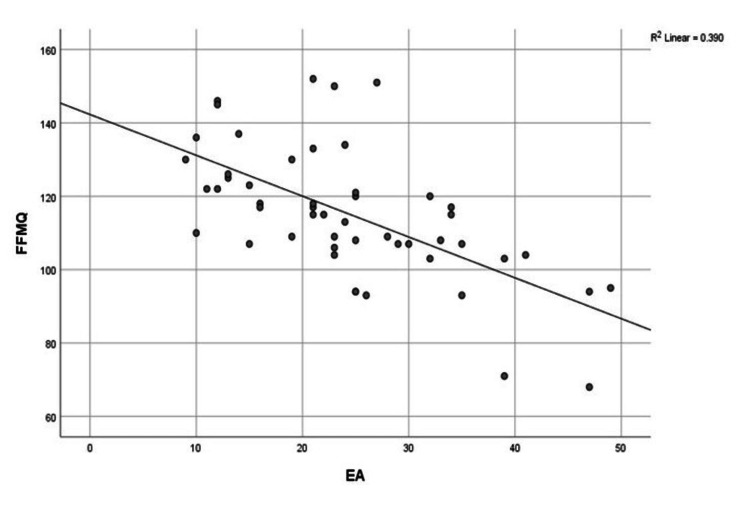
Correlation between FFMQ and EA FFMQ: Five Facet Mindfulness Questionnaire; EA: Experiential Avoidance

The correlation between quantitative data was also analyzed. It was observed that FFMQ showed a significant positive correlation with SCS and MAAS and a significant negative correlation with EA, as shown in Figures 4-6, respectively.

Correlation Between Oxidative Biomarkers

**Figure 7:**
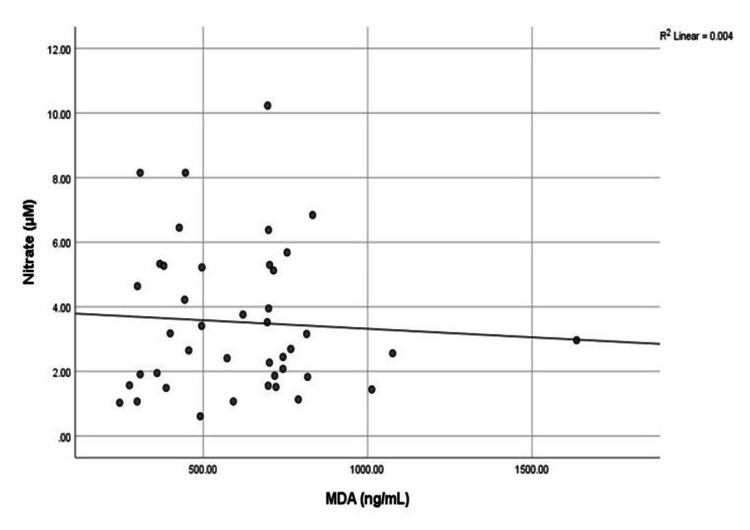
Correlation between oxidative biomarkers (serum nitrate and serum MDA) MDA: malondialdehyde

The oxidative biomarkers serum nitrate and serum MDA showed a negative correlation. However, this was not significant (Figure 7).

## Discussion

Even though there is research available in the literature regarding how HFN affects stress, burnout, and well-being, no research is available regarding the relationship between oxidative stress and psychological states after HFN training. Thus, our work adds to the body of knowledge regarding the variations in oxidative stress level markers, NO and MDA, as well as psychological aspects like positive responses to several questionnaires, including the MAAS, FFMQ, SCS, and EA, between HFN practitioners and sham controls.

The main findings of the current study are as follows: 1) There was an increase in mindfulness. Conversely, there was a decrease in the experience of avoidance. 2) The HFN group exhibited significantly higher NO and lower MDA levels than the control group did following the intervention. The oxidative stress indicators showed a negative connection in the HFN group.

The sociodemographic characteristics of both groups were observed and recorded. Furthermore, participants who engaged in HFN meditation sessions reported a noteworthy improvement in self-reported measures of psychological well-being. The PSS was a reliable instrument for determining an individual's degree of stress and was frequently employed to comprehend how different situations impact feelings and tension. The PSS evaluation for this study indicates that, before the three-month intervention, the average value was 18.79. The average score dropped to 17.18 following the session, suggesting a statistically significant decrease in stress (P-value = 0.006). This indicates a decrease in perceived stress due to the intervention of HFN meditation.

The MAAS, the most popular scale for measuring trait mindfulness, showed positive effects after the intervention. In our study, all participants in the post-experimental group (HFN meditators) obtained a total average of 54.346, SD = 13.94, while in the post-control group (non-meditators), values were 46.28, SD = 10.65, with a non-significant difference (p-value = 0.183) in both groups during pre-and post-intervention. However, earlier studies demonstrated that MAAS scores in experienced meditators (experimental group, N = 5) were 4.7 ± 0.6 as compared to inexperienced meditators (control group, N = 7) with scores of 4.1 ± 0.7 [20, 21]. Furthermore, in previous studies, a comparison was established between two groups: the yoga and comparison meditation program group (YCMP) and a control group. The MAAS values obtained for the post-YCMP and post-control groups were 4.3 ± 0.8 and 3.8 ± 0.7. The YCMP group showed a significant increase in post-intervention scores compared to pre-intervention scores [22]. However, our findings bear some resemblance to those of MacKillop and Anderson (2007), who found that in a sample of college students (of which around 10% had meditation experience), the MAAS was unrelated to meditation experience [23]. The FFMQ includes five variables; among them, only observing and not reacting to inner experience was significant; the rest of the variables are non-significant. In the post-experimental meditation group, the total mean and SD values were 123.47±14.27, while in the post-control group, the total mean and SD values were 105.95±16.66. Therefore, FFMQ is higher in the experimental group as compared to the control group between pre-and post-intervention. It suggests that individuals who scored higher in mindfulness reported indirect links to greater well-being, gratitude, pleasant affect, increased self-esteem, and self-actualization, as indicated in previous studies. Similarly, it was observed in earlier studies that FFMQ in the experimental group increased compared to the control group [24]. Another concept of mindfulness is self-compassion, as reported by Neff (2003 a, b). Mindfulness is measured by using the SCS, revealing positive outcomes in life satisfaction, optimism, and self-efficacy, along with decreased stress, anxiety, and depression. According to our study, in the post-experimental group, we noticed that out of six variables, five were significantly increased, such as self-kindness, self-judgment, common humanity, isolation, and over-identification. Therefore, self-compassion was improved in the meditator group as compared to the non-meditator group. Findings in other studies that showed SCS values for the post-YCMP and control groups were 3.2 ± 0.4 and 3.2 ± 0.4 [22, 25]. 

In the case of EA, our results for the experimental group were 19.692 (7.44), whereas for the control group, they were 29.32 (9.97). High levels of experiential avoidance, evaluated through the AAQ-II tool, were significantly negatively correlated with mindfulness. Similarly, in previous research, it was found that EA played a more significant role in explaining the levels of burnout dimensions such as “lack of development” and “overload ˮ as compared to mindfulness and self-compassion [26]. According to another study, EA decreased in the post-experimental group as compared to the post-control group after Zen meditation [24]. Our study also analyzed the correlation between different variables. As expected, FFMQ showed a significant positive correlation with SCS and MAAS and a significant negative correlation with EA.

The study revealed that the intervention of HFN meditation had a positive effect on oxidative biomarkers such as serum MDA and serum nitrate oxide levels, which were monitored to assess redox reactions in vivo. In the present investigation, a significant reduction in serum MDA level was noted after 12 weeks of intervention among the post-experimental group (426.009±117.424 ng/mL), whereas in the post-control group it was 816.37 ± 213.02 ng/mL. Furthermore, the investigation of Zen meditation in a previous study showed that the serum MDA level exhibited a notable decrease in the meditation group compared to the control group (1226.51±0.28 ng/ml vs. 1994.70±1.64 ng/ml, p<0.001) [27]. Therefore, it suggests that oxidative stress is decreased in the experimental group as compared to the control group. This aligns with findings from other studies, which have illustrated how both physical and emotional stress can elevate free radical production. Stress triggers heightened activity in the sympathetic nervous system, which disrupts cell metabolism, resulting in the formation of cytotoxic free radicals and contributing to disease progression. Practices such as yoga asanas, meditation, and pranayama serve to diminish sympathetic activity by rebalancing the autonomic nervous system towards parasympathetic dominance. Thus, following regular practice of HFN meditation can influence calmness and may be a key factor in decreased free radical formation. A previous study showed that 12 weeks of yoga intervention improved MDA levels in the yoga group (116.737±0.06 ng/ml), whereas the control group showed higher MDA levels (129.708±0.05 ng/ml) [28]. Similarly, another study conducted yoga training for 12 weeks. While the control group underwent no training intervention and continued their usual daily activities, MDA in the experimental interventional group decreased compared with the control group. The calculated p-value was found to be < 0.001, indicating statistical significance. This showed that yoga has led to a notable enhancement in MDA levels, a marker indicating oxidative stress within the body [29, 30].

In addition, another oxidative biomarker was assessed in the current study, i.e., serum NO. Nitric oxide undergoes rapid conversion into nitrite and nitrate due to its highly unstable molecules. In our present studies, we observed that after 12 weeks (three months) of intervention, the post-experimental group acutely elevated serum nitrate levels, i.e., 3.63± 2.25 µM, as compared to the post-control group, which were 3.40± 2.19 µM, respectively. Therefore, recommending even a brief HFN meditation practice can lead to an increase in serum nitrate levels. Our findings provide further evidence confirmed by the earliest study of Zen meditation; they demonstrated that the meditator group had an elevated concentration of serum nitrate + nitrite as compared to the control group (4.60 ± 0.23 µM, 2.45 ± 0.19 µM, p<0.001 [27]. Similarly, in another study, the plasma nitrate + nitrite levels were elevated in the experienced meditators group as compared to the inexperienced ones. However, there were no significant differences between nitrate or nitrite levels pre- as well as post-intervention for either experienced or inexperienced meditators throughout the study. According to previous studies, in experienced meditators, plasma nitrate was 47.91 ± 13.44 µM post-intervention, whereas inexperienced meditators plasma nitrate value was obtained (22.49 ± 2.91 µM) [21]. Therefore, serum nitrate levels were more than twice as high in the meditator group as in the non-meditator group. These findings contribute to a deeper understanding of the impact of HFN meditation on serum nitrate. According to our findings, the biological investigation showed that the oxidative stress biomarker serum MDA was reduced in the intervention group, while serum nitrate levels increased after the intervention. There was an insignificant negative correlation observed between both markers.

Strengths and limitations

The research findings highlighted the positive impact of HFN meditation on mindfulness, self-compassion, and psychological well-being in participants and showed that HFN meditation plays a crucial role in mitigating both psychological and oxidative stress responses. Future studies may be carried out by conducting larger, longitudinal studies and exploring the meditation's effects in specific clinical populations to uncover its therapeutic potential.

## Conclusions

Our study contributes to understanding the positive effects of HFN meditation on both oxidative and psychological stress, promoting relaxation, and addressing the stresses of modern lifestyles. Heartfulness meditation comprises elements such as relaxation, cleaning, and heartfulness prayer, wherein the mind and body become peaceful and connect with the heart to build self-confidence and promote the ability to balance life. To achieve the right balance in life, it is necessary to have physical energy, an enthusiastic attitude, clarity of concept, and good-quality sleep. We observed changes in biomarkers and psychological well-being, suggesting that HFN meditation holds promise as a comprehensive approach to stress management. Therefore, regular practice of HFN meditation helps to maintain a healthy lifestyle and good mental health.
